# Case report: skeletal muscle metastasis from follicular thyroid carcinoma presenting as synovial sarcoma

**DOI:** 10.3389/fonc.2023.994729

**Published:** 2023-02-10

**Authors:** Anastasia Stergioula, Evaggelos Pantelis, Theodoros Kormas, Georgios Agrogiannis

**Affiliations:** ^1^1st Department of Pathology, Medical School, National and Kapodistrian University of Athens, Athens, Greece; ^2^Radiotherapy Department, Iatropolis Clinic, Athens, Greece; ^3^Medical Physics Laboratory, Medical School, National and Kapodistrian University of Athens, Athens, Greece; ^4^Department of Orthopedic Surgery, Saint Savvas Cancer Hospital, Athens, Greece

**Keywords:** skeletal muscle metastasis, soft tissue mass, synovial sarcoma, follicular thyroid carcinoma, case report

## Abstract

Differentiated thyroid carcinomas tend to remain localized and usually are of slow progression with excellent long-term survival. The major sites of distant metastases are cervical lymph nodes, lungs and bones and the minor sites include the brain, liver, pericardium, skin, kidney, pleura and muscle. Skeletal muscle metastases from differentiated thyroid carcinoma, are exceedingly rare. In this report, a 42-year-old woman with follicular thyroid cancer that had had a total thyroidectomy and radioiodine ablation nine years ago was presented with a painful right thigh mass and negative PET/CT scan. The patient had also lung metastases during the follow-up period which were treated with surgery, chemotherapy and radiation therapy. An MRI scan of the right thigh showed a deep-seated lobulated mass with cystic regions, bleeding elements and strong heterogeneous post contrast administration enhancement. Due to the similarities in clinical manifestations and imaging features between soft tissue tumors and skeletal muscle metastases, the case was initially misdiagnosed in favor of synovial sarcoma. Histopathological, immunohistochemistry and molecular analysis of the soft tissue mass confirmed to be a thyroid metastasis and, as a result, a final diagnosis of skeletal muscle metastasis was provided. Even though the probability of a skeletal muscle metastasis from thyroid cancer approaches zero, this study aims to raise the awareness to the medical community that these events do in fact occur in the clinical setting and should be considered in the differential diagnosis of patients with thyroid carcinomas.

## Introduction

Clinically recognized thyroid carcinoma is a rare malignancy, accounting for less than 1% of human malignant neoplasms (1). Despite its low incidence, it is the most common malignancy in the endocrine system and is responsible for more deaths than all other endocrine cancers combined. The incidence shows a predominance in females with a male to female ratio of about 1:1.5 to 1:3 for most countries (1). At least 94% of all new thyroid carcinoma cases are differentiated thyroid carcinomas (DTC) that derive from the follicular epithelial cells of the thyroid gland; either papillary thyroid carcinoma (PTC) or follicular thyroid carcinoma (FTC) (1, 2). Another 5% are medullary thyroid carcinomas, neuroendocrine tumours and the remaining 1% are anaplastic thyroid carcinomas that derive from dedifferentiation of the differentiated type. The overall prognosis for patients with thyroid cancer is one of the best among all cancers. In a report of 15 698 patients in the USA, the 10-year survival rates, corrected for age and sex, were 98% for papillary, 92% for follicular, 80% for medullary, and 13% for anaplastic disease (3). Patients with DTC are managed using total thyroidectomy, radioiodine ablation and levothyroxine suppression therapy (4). Distant metastases occur during follow-up in 2.2% to 23% of patients (5–9) and are usually identified in the lungs and bones. Less frequently, they are detected in the brain, liver, pericardium, skin, kidney, pleura, and indicate poorer prognosis (8, 9). In rare cases, distant metastases to the skeletal muscles from DTC have also been observed (10). It must be noted that although the common distant metastases sites from DTC (lung and bone) usually draw significant concern, rare metastases are usually not considered or are even ignored in the clinical setting. Therefore, recognizing the patterns of rare metastases from DTC has a significant impact on the clinical decision making and prognosis of patients.

In this study, we report a skeletal muscle metastasis case from follicular thyroid carcinoma. Because of the similarities in clinical manifestations and imaging features between soft tissue tumors and skeletal muscle metastases (SMM) (11, 12), the case was initially misdiagnosed as synovial sarcoma and referred to our clinic for neo-adjuvant radiation therapy.

## Case presentation

**Figure 1:**
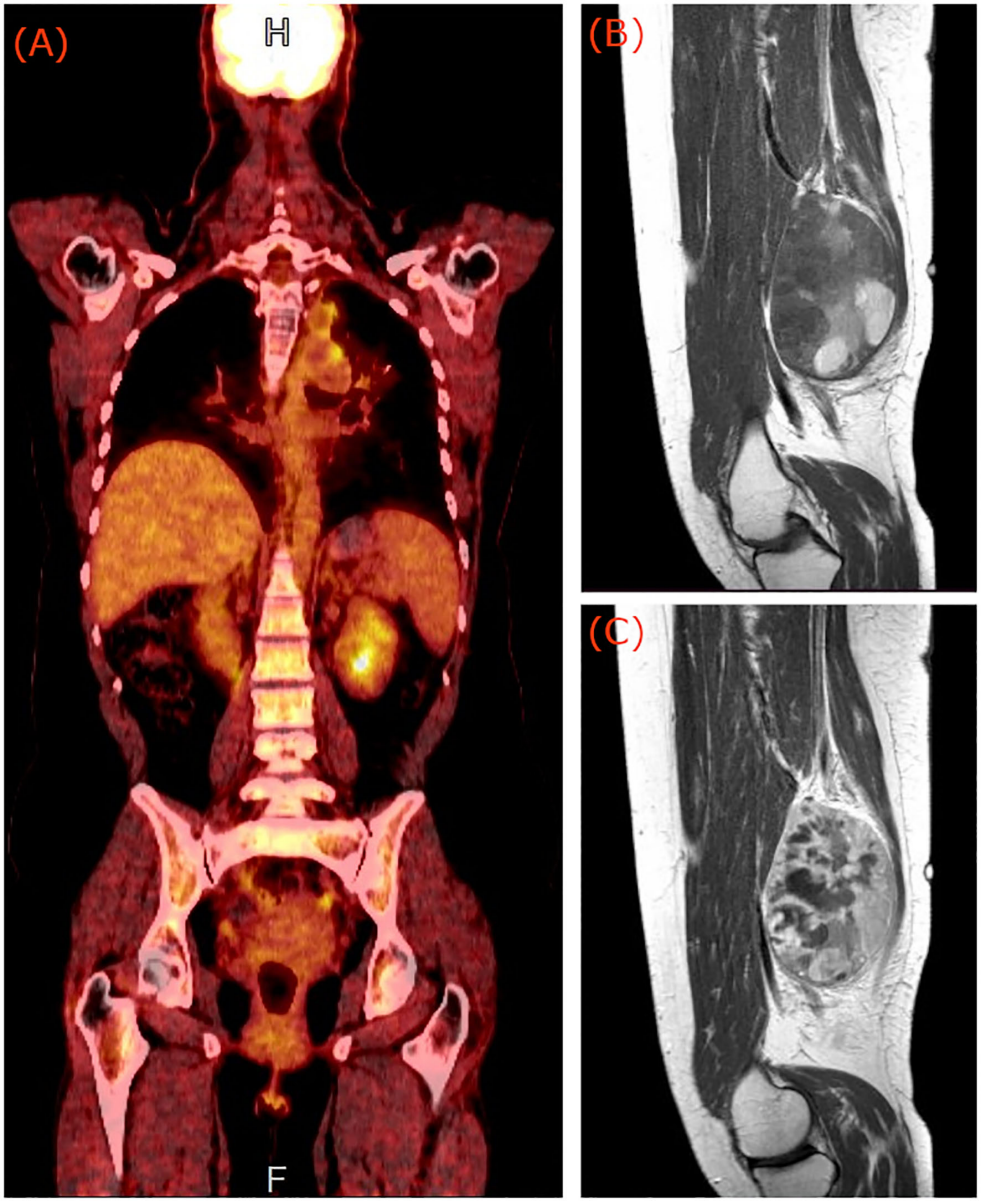
**(A)** PET/CT of the patient showing medium FDG uptake of the left hilar and mediastinum nodes. Pre **(B)** and post **(C)** contrast sagittal T1 weighted magnetic resonance images of the right thigh of the patient, showing a deep-seated lobulated mass, with cystic regions, bleeding elements and strong heterogeneous enhancement indicative of synovial sarcoma.

A 42-year-old woman was presented to our clinic with a painful soft-tissue mass of the right thigh. During clinical examination a large tender mass of the right thigh was observed. Nine years earlier, at the age of 33, the patient had undergone a total thyroidectomy for an enlarged nodule of the left thyroid lobe. The histopathological examination of the resected thyroid gland showed a follicular carcinoma and the patient received radioiodine ablation using an activity of 3.7 GBq. Serum thyroglobulin levels decreased to less than 0.04 ng/ml and the post treatment ^131^I whole body scan (^131^I-WBS) was found negative. Three years later, at the age of 36, the levels of serum thyroglobulin increased to 6.5 ng/ml and the patient received radioiodine therapy with an activity of 4.44 GBq. Although the post treatment ^131^I-WBS was found negative, the levels of serum thyroglobulin remained high. A Positron Emission Tomography/Computed Tomography (PET/CT) examination revealed a single right pulmonary metastasis which was treated with right upper lobectomy. Three years later, at the age of 39, a thorax CT scan was performed, revealing another pulmonary metastasis in the left lung which was treated with wedge resection. Moreover, on a follow-up PET/CT scan performed two years later, mediastinal lymph nodes of 2.7 cm maximum diameter and max SUV of 5.1 were detected and the patient received chemotherapy. Mediastinal lymph nodes continued to increase and the patient was treated with radiotherapy using a dose of 50 Gy in 25 fractions. On a PET/CT examination performed approximately 1 year after radiotherapy, medium FDG uptake in the lymph nodes of the mediastinum was observed and was considered indicative of residual disease (**Figure 1A**). Serum thyroglobulin was also increased and the patient received systemic therapy with tyrosine kinase inhibitor (sorafenib).

After the clinical examination of the painful soft tissue mass in conjunction with the absence of other regions with pathological FDG uptake in the last PET/CT scan, an MRI examination of the right thigh was requested. Thigh MRI revealed a (10 × 8.5) cm^2^ lobulated mass, situated deep in the posterior compartment with cystic regions, bleeding elements, fluid-fluid levels, and post contrast administration strong heterogeneous enhancement (**Figures 1B, C**). These imaging findings are indicative of synovial sarcoma (12).

**Figure 2:**
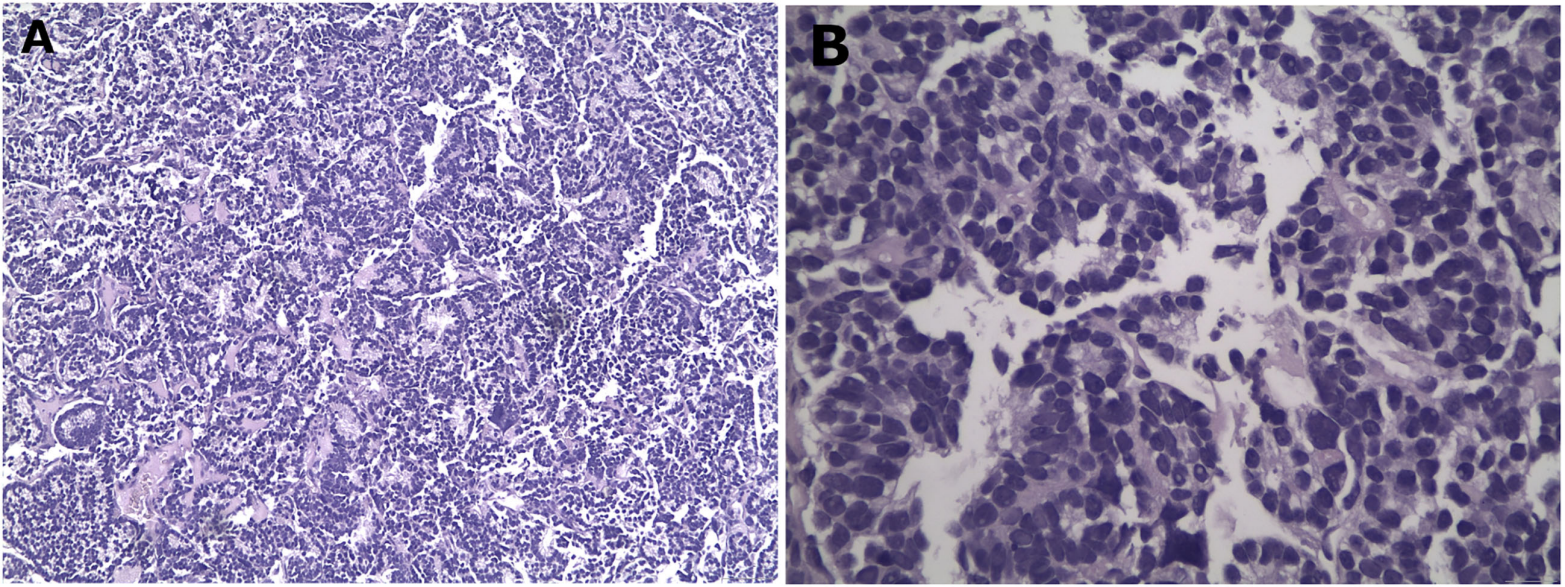
Representative histological images from the biopsy specimen. Low (40x) **(A)** and high (400x) **(B)** power views showing solid and acinar growth pattern, poor differentiation, eosin – hematoxylin stain.

**Figure 3:**
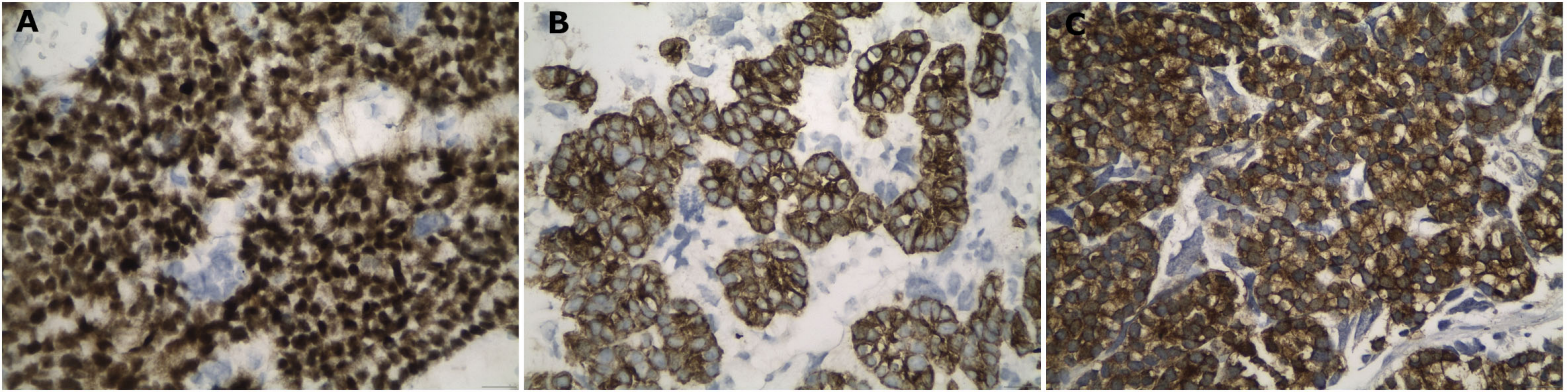
High power views of the histological images, showing strong nuclear positivity for TLE-1 **(A)**, strong positivity against pancytokeratin **(B)** and, finally, strong immunoreactivity for thyroglobulin proving the origin from the thyroid gland **(C)**. Immunohistochemical peroxidase staining, diaminobenzidine as chromogen.

A biopsy of the soft tissue mass was performed to confirm imaging findings. Histologic examination revealed a neoplastic population of high cellularity, poorly differentiated with a pseudogladular and focally solid growth pattern (**Figure 2**). The cellular elements showed medium-sized, deep-colored subround nuclei with moderate nuclear atypia. Extensive areas of necrosis were found in approximately 30% of the total biopsy material. Immunohistochemistry showed strong nuclear positivity for TLE1. The neoplastic cells which reacted focally with antibodies directed against CD99, were reactive for the cell markers AE1/AE3 and negative for the markers S100, CD34 and MyoD1, supporting the diagnosis of synovial sarcoma (**Figures 3A, B**). A molecular examination for synovial sarcoma was also requested. The molecular examination for SYT-SSX translocation was negative. Hence further immunohistochemistry examination was performed using additional stains, revealing a thyroid transcription factor 1 and thyroglobulin-positive cells, suggestive of a metastatic tumor originating from thyroid tissue (**Figure 3C**).

Following the correct diagnosis, the thigh metastasis was treated using surgical resection and pre-operative radiotherapy to a dose of 50 Gy in 25 fractions.

## Discussion

Differentiated thyroid carcinomas tend to remain localized and are usually of slow progression with excellent long-term survival. Distant metastases occur during follow-up with a probability of up to 23% (5–9). The major sites of the distant metastases of DTC are the lung and bone. The minor sites include the brain, liver, pericardium, skin, kidney, pleura and muscle and indicate a poorer prognosis (8, 9). Hematogenous metastasis of follicular or papillary thyroid cancer to skeletal muscle is exceptionally rare. It has been hypothesized that skeletal muscle is a hostile environment for the retention and proliferation of cancer cells, due to muscle motion, unadapted muscle pH, and the muscle’s ability to remove tumor-produced lactic acid (13). In a recent literature search, 44 published international papers were found reporting 58 cases of PTC or FTC skeletal muscle metastasis in 45 patients in 110 years; from 1907 to 2017 (10). Analysis of epidemiological data showed that the probability of detecting a skeletal metastasis is 4 in every 1 billion people. The most frequent DTC metastatic muscle was the gluteus. Most of the muscle metastasis cases were caused by PTC, which is the most common differentiated thyroid carcinoma. Metastatic tumors in the skeletal muscle were found to negatively impact the survival of patients with PTC or FTC. It should be noted, however, that while incidence of a muscle metastasis from DTC is approximately zero, this phenomenon does happen in the clinical setting.

The measurable serum thyroglobulin level and ^131^I-WBS are commonly used for the follow-up of patients with thyroid cancer. In the presence of elevated thyroglobulin and negative ^131^I-WBS scan, PET/CT imaging has been proven a useful tool for detecting metastatic lesions from DTCs (14). The standard torso imaging PET/CT scan, however, covers the base of skull to mid-thigh, or upon indication, includes also the brain in the same scan (cranially extended torso imaging: from the top of the head to mid-thigh) (15). Therefore, possible metastases located lower than the mid-thigh level will not be imaged. In the reported case, diagnosis was based on findings from a cranially extended PET/CT scan and an MRI scan of the right thigh. While the PET/CT scan revealed two medium FDG uptake nodes in the left hilar and mediastinum, it failed to localize the metastasis in the right thigh of the patient which was located near the joint of the knee. The MRI scan showed a deep-seated lobulated mass, with cystic regions, bleeding elements and strong heterogeneous post contrast administration enhancement. Due to the similarities in clinical manifestations and imaging features between soft tissue tumors and skeletal muscle metastases (12), the case was initially misdiagnosed in favor of synovial sarcoma.

Synovial sarcoma displays a diverse clinicopathological spectrum. Even though it is an aggressive sarcoma, it is amenable to treatment modalities, including chemotherapy. Hence, its correct identification which relies on pathological investigation is vital. While the histopathological and immunohistochemistry examination confirmed the imaging findings for synovial sarcoma, the molecular examination for SYT-SSX translocation was negative and therefore further immunohistochemistry examination was performed, using additional stains. The immunohistochemistry examination confirmed that the observed lesion was a metastatic tumor originating from the follicular thyroid carcinoma.

In conclusion, this study presents a rare case of a skeletal muscle metastasis from FTC. We propose that in the presence of elevated thyroglobulin and negative ^131^I -WBS, a whole-body PET/CT scan should be acquired. Histopathological, immunohistochemistry and molecular examination of biopsy samples should be incorporated to confirm imaging findings in order to reach the final diagnosis and prompt therapeutic strategy. Reporting these uncommon distant metastases of FTC is essential for a better understanding of the aggressive pathological behavior of an indolent cancer type and management of future cases.

## Data availability statement

The original contributions presented in the study are included in the article/supplementary material. Further inquiries can be directed to the corresponding author.

## Ethics statement

Ethical review and approval was not required for the study on human participants in accordance with the local legislation and institutional requirements. The patients/participants provided their written informed consent to participate in this study.

## Author contributions

AS and TK are the lead clinicians in the management of patient’s thigh metastasis. GA is the clinician performing the histopathological and immunohistochemistry examination. EP is the medical physicist responsible for radiological image analysis and radiation therapy planning. All authors contributed to the article and approved the submitted version.
